# A subset of MMR‐proficient colon cancers responds to neoadjuvant immunotherapy

**DOI:** 10.1002/1878-0261.70178

**Published:** 2025-11-30

**Authors:** Eleonora Piumatti, Giovanni Germano, Pietro Paolo Vitiello, Alberto Bardelli

**Affiliations:** ^1^ Department of Oncology, Molecular Biotechnology Center University of Torino Turin Italy; ^2^ IFOM ETS–The AIRC Institute of Molecular Oncology Milan Italy; ^3^ Department of Medical Biotechnologies and Translational Medicine University of Milano Italy

**Keywords:** chromosomal instability, circulating tumor DNA, Colon cancer, MMR‐proficient, neoadjuvant immunotherapy, tumor microenvironment

## Abstract

Mismatch repair‐proficient (pMMR) colorectal cancers (CRC) have long been considered nonresponsive to immune checkpoint blockade (ICB), in contrast to their mismatch repair‐deficient (dMMR) counterparts. Recent evidence indicates that neoadjuvant immunotherapy can be used to treat pMMR CRC before surgery, potentially reducing postoperative relapse. Tan *et al*. report results from the NICHE‐2 trial, which achieved a 26% response rate in early‐stage pMMR colon cancer (CC) patients. Molecular studies show that despite low tumor mutational burden (TMB), responders exhibit higher chromosomal instability (CIN), TP53 mutations, and enrichment of proliferative and cell‐cycle signatures, associated with higher density of Ki‐67^+^ tumor and CD8^+^ T cells. In contrast, nonresponders display metabolic and stromal reprogramming, enhanced TGF‐β signaling, and immune exclusion. Circulating tumor DNA (ctDNA) clearance correlated with pathological response and long‐term disease‐free survival postsurgery. While the biological and molecular determinants underlying the response rates observed in the NICHE‐2 trial remain to be fully elucidated, the work by Tan et al. suggests that biomarker‐guided neoadjuvant immunotherapy could represent a valuable strategy to achieve pathological responses in early‐stage pMMR CC, despite its clinical relevance requiring further evaluation.

## Graphical abstract

Tan *et al*. reveal that a distinct subset of early‐stage pMMR colon cancers can respond to neoadjuvant immunotherapy. In the NICHE‐2 trial, responders (26%) were characterized by chromosomal instability, TP53 mutations, and proliferative cell‐cycle programs, whereas nonresponders showed metabolic and stromal reprogramming with TGF‐β‐driven immunosuppressive microenvironments. These features highlight emerging biomarkers to identify pMMR tumors potentially amenable to immunotherapy.

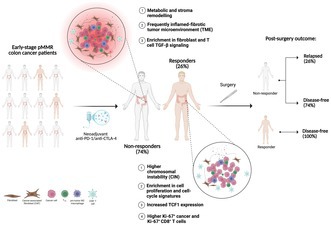

**Abbreviations**
CC: colon cancer
CIN: chromosomal instability
CRC: colorectal cancer
ctDNA: circulating tumor DNA
dMMR: mismatch repair‐deficient
ICB: immune checkpoint blockade
pMMR: mismatch repair‐proficient
TMB: tumor mutational burden
TME: tumor microenvironment

Approximately 15% of stages I–III colorectal cancers (CRC) are DNA mismatch repair‐deficient (dMMR) while the remaining 85% are mismatch repair‐proficient (pMMR). This distinction is clinically relevant, as responsiveness to immune checkpoint blockade (ICB) differs dramatically between the two groups [1]. dMMR tumors display a high tumor mutational burden (TMB), neoantigen load, and sensitivity to immunotherapy, while pMMR tumors are generally refractory [1]. Since the first NICHE trial exploring neoadjuvant ICB in early‐stage colon cancer (CC) [2], the most remarkable results have been observed in dMMR tumors, which achieved major pathological response rates exceeding 90% [3, 4]. However, unexpectedly, meaningful activity was also detected in locally advanced pMMR CC, with 27% showing pathological responses in the same study [2], prompting further exploration of this strategy also in this subset of patients [5].

In this scenario, the study by Tan *et al*. provides additional evidence that a subset of locally advanced pMMR CC can respond to neoadjuvant ICB. The phase II NICHE trial (NCT03026140) enrolled 31 patients with pMMR early‐stage CC, identified based on radiologic staging. In this population, the authors report that ipilimumab plus nivolumab treatment prior to surgery elicits a response rate of 26%, including one clinical complete response and seven pathological responses (of which three are pathological complete responses). Importantly, none of the responders experienced disease recurrence, remaining disease‐free for up to 72 months after surgery, underscoring the potential for durable benefit in responding patients (Fig. 1). Among the key findings, the presence of circulating tumor DNA (ctDNA) after treatment strongly correlated with clinical outcome (Table 1). Nearly all responders became ctDNA‐negative and remained disease‐free, whereas patients who eventually relapsed were ctDNA‐positive before surgery (Table 1). These findings suggest that ctDNA clearance is a promising, minimally invasive genomic biomarker to monitor neoadjuvant treatment response and postoperative relapse risk, although this application remains to be formally validated in larger cohorts.

**Fig. 1:**
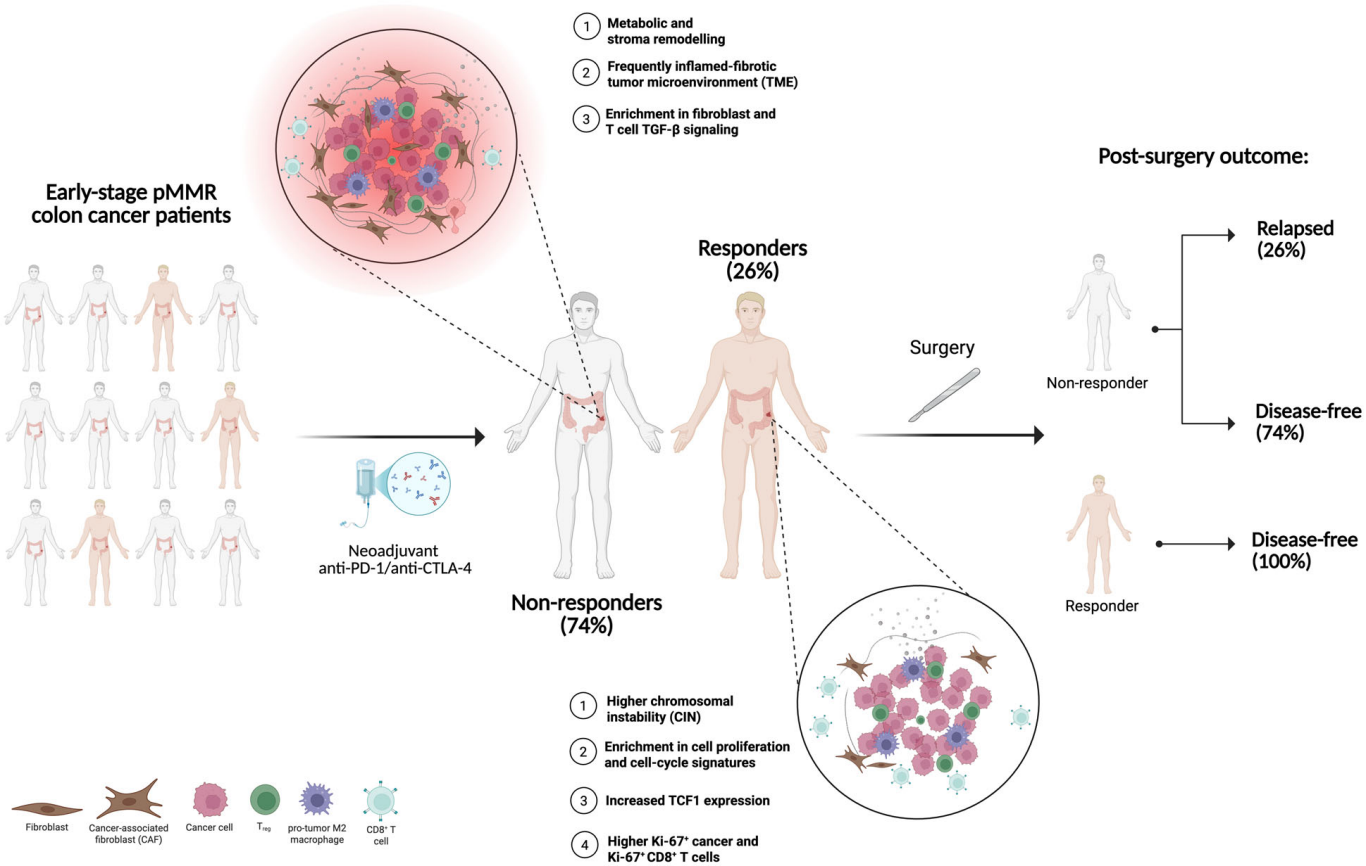
Early‐stage pMMR Colon Cancer Response to Neoadjuvant Immunotherapy. Neoadjuvant immune checkpoint blockade in early‐stage pMMR colon cancers (NICHE‐2 trial) elicits responses in a subset of tumors. Responders (26%) exhibit chromosomal instability, TP53 mutations, and enrichment of proliferative and cell‐cycle–related programs, whereas non‐responders (74%) show metabolic and stromal reprogramming with TGF‐β‐driven immunosuppressive microenvironments. Postsurgery outcomes indicate that responders remain disease‐free, whereas nonresponders are at higher risk of recurrence. Created with BioRender.com.

**Table 1:** ctDNA dynamics in responder and nonresponder early‐stage pMMR CC patients. Number and percentage of patients with ctDNA‐positive or ctDNA‐negative status before and after neoadjuvant immunotherapy, including ctDNA clearance, stratified by response (responders vs non‐responders) and postoperative outcome (disease‐free vs recurrence). CC, colon cancer; cCR, complete clinical response; ICB, immune checkpoint blockade; MPR, major pathological response; NR, nonresponders; pCR, pathological complete response; pMMR, mismatch repair‐proficient; PR, pathological response; R, responders.

	Responders (R) to neoadjuvant ICB		Non‐responders (NR) to neoadjuvant ICB	
Total patients (31)	8/31 (26%)^a^		23/31 (74%)	
	**Baseline**	**Presurgery**	**Baseline**	**Presurgery**
ctDNA‐positive	6	/	20	19
ctDNA‐negative	2	7^b^ (2 at baseline + 5 cleared)	3	4 (3 at baseline + 1 cleared)
ctDNA cleared [ctDNA‐positive baseline ➔ ctDNA‐negative presurgery]	5/6 (83%)		1/20 (5%)	
Postneoadjuvant ctDNA status in disease‐free patients	8/8 (100%)		17/23 (74%)	
	**ctDNA‐positive**	**ctDNA‐negative**	**ctDNA‐positive**	**ctDNA‐negative**
	1	7	13	4
Postneoadjuvant ctDNA status in relapsed patients	/		6/23 (26%)	
	**ctDNA‐positive**	**ctDNA‐negative**	**ctDNA‐positive**	**ctDNA‐negative**
	/	/	6	/
Postsurgical ctDNA status^c^	**ctDNA‐positive**	**ctDNA‐negative**	**ctDNA‐positive**	**ctDNA‐negative**
	/	7^b^	2	21

a 26% patients responded to neoadjuvant immunotherapy: 1 cCR, 7 PR (6/7 MPR, 3/6 pCR).

b For 1 patient not available (NA).

c Three weeks postsurgery.

Genomic analysis of pMMR tumors revealed that pathological responses occurred independently of TMB. Instead, chromosomal instability (CIN) emerged as a major correlate of benefit, likely reflecting the higher prevalence of whole‐genome duplication events in these tumors (Fig. 1). Moreover, the enrichment of responders for a TP53‐mutated and KRAS‐wild‐type background is consistent with the well‐described suppression of immune pathways and reduced immune infiltration driven by KRAS alterations (Fig. 1) [6, 7].

At the transcriptomic level, classical immune‐related signatures did not predict response. Instead, responders were characterized by enrichment of proliferative and cell‐cycle programs, including a higher expression of TCF1 (Fig. 1). Imaging mass cytometry further corroborated these findings, revealing increased Ki‐67^+^ tumor cells and Ki‐67^+^ CD8^+^ T cells (Fig. 1). Together, these results suggest that proliferative features may facilitate effective immune engagement. The authors then extended the analysis beyond the immune compartment and dissected additional modulators of response within the tumor microenvironment (TME); these included stromal architecture, metabolic programs, and the proliferative state of both tumor and immune cells. These analyses did not lead to findings of immediate clinical relevance. However, this study highlighted several features that may predict benefit, including proliferative and cell‐cycle‐related signatures in responders, and metabolic and stromal reprogramming in nonresponders (Fig. 1). Specifically, nonresponders exhibited enhanced fatty‐acid metabolism, mTOR signaling, glycolysis, and oxidative phosphorylation following neoadjuvant treatment, together with increased matrix remodeling and an inflamed‐fibrotic TME dominated by TGF‐β signaling, which is known to exclude T cells and mediate ICB resistance (Fig. 1) [8].

These results indicate that stromal features, TGF‐β and KRAS status, and metabolic pathways may influence neoadjuvant response in early‐stage pMMR CC and could represent actionable vulnerabilities in nonresponders to potentially enhance ICB efficacy.

Collectively, the encouraging response rates observed in this trial suggest that a significant subset of nonmetastatic pMMR CC can receive benefit from immunomodulation, and that genomic, transcriptomic, metabolic, and immunological biomarkers, as well as ctDNA surveillance, could be used to refine patient selection and monitor treatment efficacy [2]. However, despite the promising results of this and other studies, neoadjuvant immunotherapy in early‐stage pMMR CC still faces several challenges and many open questions remain. As acknowledged by the authors, accurate radiological staging of early‐stage CC is difficult [9], complicating the selection of suitable candidates and possibly leading to overtreatment. Of note, 45% of the patients enrolled in this trial, including 4/7 responders, presented a stages I–II tumor at the radiological staging, which would have probably not required any additional treatment besides surgery by clinical practice. In addition, since surgery is generally well‐tolerated and remains the standard of care in early‐stage CC, the potential benefits of neoadjuvant therapy must be balanced against its additional risks, including treatment‐related toxicities that could delay surgery or affect long‐term quality of life, as reflected by the 11% incidence of persistent endocrine toxicities reported in the NICHE‐2 trial [3]. This scenario differs from rectal cancer, where surgery and radiotherapy often have a profound impact on functional outcomes and quality of life, making a neoadjuvant immunotherapy approach much more appealing with the aim of pursuing a nonoperative management. In CC, particularly for pMMR cases, the added clinical benefit of neoadjuvant immunotherapy over upfront surgery remains an open and actively debated question, unless supported by solid evidence demonstrating meaningful reduction in postoperative relapse over the standard treatment.

Other areas of active development are emerging in the challenge to increase the immune sensitivity of pMMR tumors. Microbiota modulation represents a promising strategy, given its role in shaping the response to ICB in metastatic pMMR CRC [10]. In parallel, next‐generation immunotherapy combinations are under investigation in early‐stage resectable CRC to overcome resistance to PD‐1/CTLA‐4‐based therapy. These include LAG‐3 inhibition with relatlimab in the NICHE‐3 trial [11] and the novel Fc‐enhanced anti‐CTLA‐4 antibody botensilimab in the NEST‐1 trial [5]. Collectively, these approaches may extend neoadjuvant immunotherapy benefit to more early‐stage pMMR CC patients if validated in currently ongoing clinical trials.

In conclusion, the work by Tan *et al*. reshapes current assumptions on early‐stage pMMR CC, by showing that even tumors traditionally considered immunotherapy‐refractory harbor a subset of patients who achieve durable pathological responses, with the potential to meaningfully reduce the risk of relapse. The study of Tan *et al*. provides a molecular framework integrating genomic, transcriptomic, and immune profiling, to guide precision immunotherapy in early‐stage pMMR CC. This approach will require prospective validation in larger cohorts and a longer follow‐up to determine whether patients achieving a pathological response, including ctDNA clearance, remain recurrence‐free over time. Nonetheless, this study paves the way for integrating multiple molecular determinants of response to neoadjuvant ICB in immune‐refractory early‐stage pMMR CC, possibly marking a shift from resistance to durable immune control in non‐metastatic pMMR tumors.

## Conflict of interest

Giovanni Germano and Alberto Bardelli are cofounders and shareholders of NeoPhore. Alberto Bardelli reports receipt of grants/research support from NeoPhore, AstraZeneca and Boehringer Ingelheim and honoraria/consultation fees from Guardant Health. Alberto Bardelli is a stock shareholder of Kither Biotech. Alberto Bardelli is an advisory board member for NeoPhore. The remaining authors declare no conflict of interest.

## Author contributions

AB and EP conceived the commentary. EP, GG, and PPV wrote the draft. AB critically reviewed the final version.
